# The ORF3a protein of SARS-CoV-2 induces apoptosis in cells

**DOI:** 10.1038/s41423-020-0485-9

**Published:** 2020-06-18

**Authors:** Yujie Ren, Ting Shu, Di Wu, Jingfang Mu, Chong Wang, Muhan Huang, Yang Han, Xue-Yi Zhang, Wei Zhou, Yang Qiu, Xi Zhou

**Affiliations:** 1 Center for Precision Translational Medicine of Wuhan Institute of Virology & Guangzhou Women and Children’s Medical Center, Guangzhou Women and Children’s Medical Center, Guangzhou, Guangdong 510120 China; 2 State Key Laboratory of Virology, Wuhan Institute of Virology, Center for Biosafety Mega-Science, Chinese Academy of Sciences (CAS), Wuhan, Hubei 430071 China; 3 Center for Precision Translational Medicine of Wuhan Institute of Virology & Guangzhou Women and Children’s Medical Center, Wuhan Institute of Virology, CAS, Wuhan, Hubei 430071 China; 4 Joint Laboratory of Infectious Diseases and Health, Wuhan Institute of Virology & Wuhan Jinyintan Hospital, CAS, Wuhan, Hubei 430023 China; 5 Center for Translational Medicine, Wuhan Jinyintan Hospital, Wuhan, Hubei 430023 China; 6 State Key Laboratory of Virology, College of Life Sciences, Wuhan University, Wuhan, Hubei 430072 China; 7 University of Chinese Academy of Sciences, Beijing, 100049 China

**Keywords:** Cell death and immune response, Immune cell death

Severe acute respiratory syndrome coronavirus-2 (SARS-CoV-2) has caused the ongoing pandemic of Coronavirus Disease 2019. SARS-CoV-2 belongs to the genus *Betacoronavirus* of the *Coronaviridae* family, which includes SARS-CoV and Middle East respiratory syndrome coronavirus.^1,2^ Coronavirus-encoded accessory proteins play critical roles in virus–host interactions and the modulation of host immune responses, thereby contributing to coronaviral pathogenicity via different strategies.^3^ However, the functions of SARS-CoV-2-encoded accessory proteins are not well understood. Apoptosis is a predominant type of programmed cell death, and has been recognized as an important host antiviral defense mechanism that controls viral infection and regulates the inflammatory response.^4,5^ Previous studies have reported that the SARS-CoV-encoded accessory protein ORF3a can induce apoptosis in cells,^6,7^ leading to the question of whether SARS-CoV-2 ORF3a also has pro-apoptotic activity. Here, we investigated the potential apoptosis-inducing activity of SARS-CoV-2 ORF3a in different cell lines and compared the pro-apoptotic activities of SARS-CoV-2 ORF3a with those of SARS-CoV ORF3a using the same system.

We sought to determine whether SARS-CoV-2 ORF3a can induce apoptosis using annexin V-fluorescein 5-isothiocyanate(FITC)/propidium iodide (PI) double staining in cultured HEK293T, HepG2, and Vero E6 cells. We found that annexin V and PI staining was significantly increased in cells expressing SARS-CoV-2 ORF3a compared with that in control cells (Fig. 1a). Moreover, the quantified data based on measuring the apoptosis rate also confirmed the pro-apoptotic activity of ORF3a in different cell lines (Fig. 1b). Furthermore, we examined activated caspase-3, a marker of caspase-dependent apoptosis, by flow cytometry and found that the percentage of cells with activated caspase-3 was significantly elevated in the presence of ORF3a (Fig. 1c). These results show that SARS-CoV-2 ORF3a can efficiently induce apoptosis in cells.

**Fig. 1:**
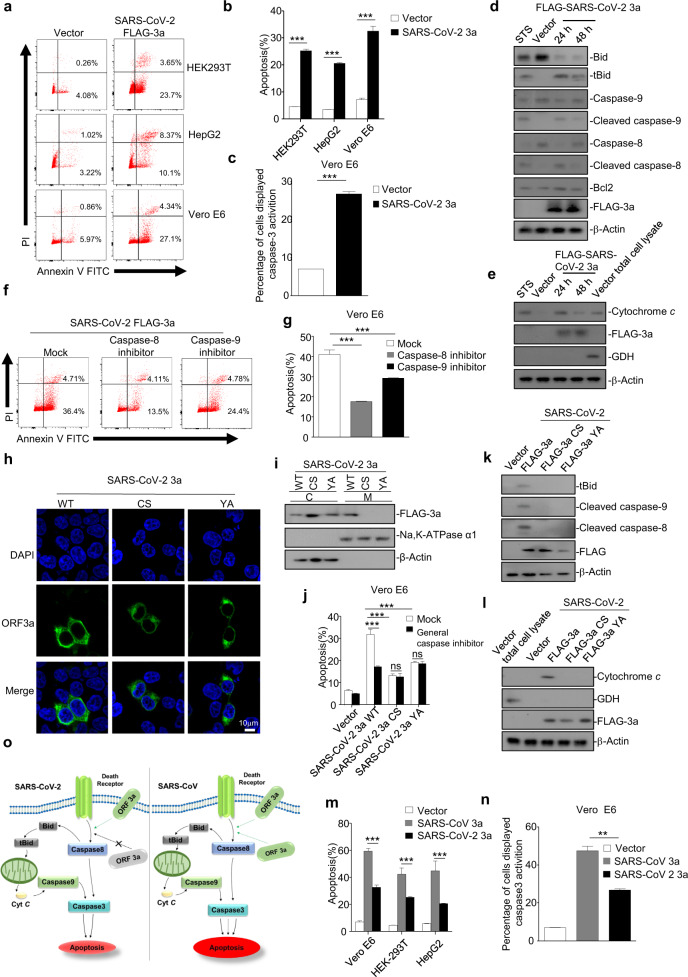
**a**, **b** HEK293T, HepG2, and Vero E6 cells were transfected with FLAG-SARS-CoV-2 ORF3a. After 24 h, cells were stained with annexin V-fluorescein 5-isothiocyanate (FITC)/propidium iodide (PI) for flow cytometric analysis (**a**), and the percentage of apoptotic cells was measured (**b**). **c** Vero E6 cells were transfected with FLAG-SARS-CoV-2 ORF3a. After 24 h, cells were stained with caspase-3/7 green detection reagent for fluorescence analysis, and the percentage of cells displaying caspase-3 activation was measured. **d** HEK293T cells were transfected with empty vector or FLAG-SARS-CoV-2 ORF3a. After 12 and 24 h, cells were subjected to western blotting analysis using the indicated antibodies. Cells treated with STS for 5 h were used as a positive control. STS, staurosporine. **e** HEK293T cells transfected with empty vector or FLAG-SARS-CoV-2 ORF3a for 12 and 24 h, or cells treated with STS for 5 h, were collected and the mitochondria were separated via gradient centrifugation. Cell lysates without mitochondria were subjected to western blotting using the indicated antibodies. The total cell lysates within intact mitochondria were used as positive control. GDH, glutamate dehydrogenase. **f**, **g** Vero E6 cells were transfected with FLAG-SARS-CoV-2 ORF3a in the presence of DMSO, caspase-8 inhibitor, or caspase-9 inhibitor. After 24 h, cells were stained with annexin V-FITC/PI for flow cytometric analysis (**f**), and the percentage of apoptotic cells was measured (**g**). **h** HEK293T cells were transfected with FLAG-SARS-CoV-2 ORF3a and its mutants (CS and YA). After 24 h, cells were stained with a mouse anti-FLAG antibody and Alexa-488 conjugated anti-mouse IgG for immunofluorescence. Scale bar, 10 μM. **i** HEK293T cells were transfected with FLAG-SARS-CoV-2 ORF3a and its mutants (CS and YA). After 24 h, cells were collected and the membrane and plasma proteins were separately extracted for western blotting. **j** Vero E6 cells were transfected with FLAG-SARS-CoV-2 ORF3a mutants (CS and YA) and treated with DMSO or a general caspase inhibitor. After 24 h, cells were stained with annexin V-FITC/PI for flow cytometric analysis, and the percentage of apoptotic cells was measured. **k**, **l** HEK293T cells were transfected with vector or FLAG-SARS-CoV-2 ORF3a and its mutants (CS and YA). After 24 h, cells were collected and the membrane and plasma proteins were separately extracted for western blotting (**k**). To examine levels of cytochrome *c* in the cytosol, mitochondria were separated via gradient centrifugation, and cell lysates without mitochondria were subjected to western blotting (**l**). **m** HEK293T, HepG2, and Vero E6 cells were transfected with vector, FLAG-SARS-CoV ORF3a, or FLAG-SARS-CoV-2 ORF3a. After 24 h, cells were stained with annexin V-FITC/PI for flow cytometric analysis, and the percentage of apoptotic cells was measured. **n** Vero E6 cells were transfected with vector, FLAG-SARS-CoV ORF3a, or FLAG-SARS-CoV-2 ORF3a. After 24 h, caspase-3 activation was detected by using caspase-3/7 green detection reagent, and the percentage of cells displaying caspase-3 activation was measured. **o** Left, the pro-apoptotic activity of SARS-CoV-2 ORF3a requires the membrane association of ORF3a. Right, membrane association is involved but not essential for the pro-apoptotic activity of SARS-CoV ORF3a, and SARS-CoV ORF3a can induce apoptosis in a membrane-independent manner. SARS-CoV-2 ORF induces apoptosis in a lesser extent than that of SARS-CoV ORF3a. **p < 0.01, ***p < 0.001 by two-tailed Student’s *t* test

To determine the mechanism through which SARS-CoV-2 ORF3a induces apoptosis, activation of the apoptosis cascade in HEK293T cells expressing ORF3a was examined by western blotting, probing for some apoptosis pathway components at 24 and 48 h post transfection. Cells treated with staurosporine, an apoptosis inducer, were used as a positive control. SARS-CoV-2 ORF3a induced the cleavage/activation of caspase-8, whereas Bcl-2 expression levels were not affected (Fig. 1d). The cleavage/activation of caspase-8 is recognized as a hallmark of the extrinsic apoptotic pathway, whereas Bcl-2 plays an important role in initiation of the intrinsic pathway.^8^ Moreover, we found that the levels of truncated Bid (tBid), cleaved caspase-9, and cytochrome *c* were elevated in the presence of SARS-CoV-2 ORF3a (Fig. 1e), and either a caspase-8 or caspase-9 inhibitor significantly suppressed SARS-CoV-2 ORF3a-induced apoptosis (Fig. 1f, g). Thus, our results imply that SARS-CoV-2 ORF3a can induce apoptosis via the extrinsic pathway, in which activated caspase-8 cleaves Bid to tBid and in turn induces the release of mitochondrial cytochrome *c*, resulting in apoptosome formation and caspase-9 cleavage/activation.

We next sought to examine the relationship between the membrane association and pro-apoptotic activity of SARS-CoV-2 ORF3a. As previously reported, SARS-CoV ORF3a is a transmembrane protein that contains several conserved motifs including a cysteine-rich motif (a.a.127–133), tyrosine-based sorting motif (YXXΦ; a.a.160–163), and diacidic EXD motif (a.a. 171–173), and these domains regulate the subcellular location of SARS-CoV ORF3a and play important roles in SARS-CoV ORF3a infection, inducing apoptosis.^9,10^ SARS-CoV-2 ORF3a shares 73% amino acid homology with its counterpart in SARS-CoV, and the cysteine-rich and YXXΦ motifs are conserved but the EXD motif was found to be changed to SGD in SARS-CoV-2 ORF3a (Fig. S1a). Thus, we constructed two mutant ORF3a proteins by mutating C130/133 of the cysteine-rich motif to S (SARS-CoV-2 ORF3a-CS) or Y160 of the YXXΦ motif to A (SARS-CoV-2 ORF3a-YA). The immunofluorescence assays showed that wild-type ORF3a of SARS-CoV-2 (ORF3a-WT) localized to the plasma membrane with punctate cytoplasmic staining, whereas ORF3a-CS and ORF3a-YA exhibited more cytoplasmic localization (Figs. 1h and S1b). The results of cytosol-membrane fractionation assays showed that whereas ORF3a-WT was present in both cytosol and membrane fractions, either ORF3a-CS or ORF3a-YA was absent in the membrane fraction (Figs. 1i and S1c). Moreover, we found that ORF3a-CS or ORF3a-YA showed minimal apoptosis-inducing and caspase-3-activiting activity in cells in the presence or absence of z-VAD-fmk, a general caspase inhibitor (Figs. 1j and S1d). In addition, ORF3a-CS or ORF3a-YA failed to induce the cleavage of Bid, caspase-8, and caspase-9 or the release of cytochrome *c* (Fig.1k, l). These results indicate that membrane association is required for the pro-apoptotic activity of SARS-CoV-2 ORF3a.

To investigate if there is any difference between the pro-apoptotic activities of ORF3a proteins of SARS-CoV-2 and SARS-CoV, we examined the membrane association and apoptosis-induction ability of SARS-CoV ORF3a. SARS-CoV ORF3a variants were generated by mutating C127/130/133 to S (SARS-CoV ORF3a-CS) in the cysteine-rich motif, Y160 to A (SARS-CoV ORF3a-YA) in the diacidic motif, and 171E/173D to A (SARS-CoV ORF3a-DE) in the EXD motif. We found that SARS-CoV ORF3a-CS and ORF3a-YA mutants were unable to associate with membranes or distribute in membrane fractions, whereas ORF3a-DE still showed membrane association similar to that observed for ORF3a-WT (Fig. S2a, b). Although the ORF3a-CS and ORF3a-YA mutants showed significantly lower apoptosis-inducing and caspase-3-activating capacities than WT ORF3a, they kept some pro-apoptotic activities compared with those of the negative control (vector), which could be further inhibited by z-VAD-fmk treatment (Fig. S2c, d). Moreover, the cleavage of Bid, caspase-8, and caspase-9 or the release of cytochrome *c* was apparently reduced but not eliminated in cells expressing SARS-CoV ORF3a-CS or SARS-CoV ORF3a-YA (Fig. S2e, f). These results indicate that unlike that in SARS-CoV-2 ORF3a, the membrane-association feature is involved in but not essential for the pro-apoptotic activity of SARS-CoV ORF3a (Fig. S3), suggesting that the two ORF3a proteins from different coronaviruses use different strategies to induce apoptosis.

We then sought to compare pro-apoptotic activities between these two coronaviral proteins. Our results showed that compared with those with SARS-CoV-2 ORF3a, SARS-CoV ORF3a expression induced higher levels of apoptosis in Vero E6, HEK293T, and HepG2 cells (Fig. 1m). Consistently, the caspase-3 activation level in SARS-CoV ORF3a-expressing cells was significantly higher than that in cells expressing SARS-CoV-2 ORF3a (Fig. 1n). Therefore, our findings show that SARS-CoV-2 ORF3a has relatively weaker pro-apoptotic activity than SARS-CoV ORF3a. Differences in the pro-apoptotic mechanism and relative strength probably contribute to the differences in pathogenicity between these two coronaviruses (Fig. 1o). Indeed, SARS-CoV-2 has been generally believed to be less virulent than SARS-CoV, and the diminished pro-apoptotic activity of SARS-CoV-2 ORF3a is probably associated with reduced apoptosis-mediated antiviral defence in infected cells. These properties probably confer certain advantages for SARS-CoV-2 in that infection can be relatively mild or even asymptomatic during early stages, thus allowing the virus to spread more widely.

In summary, the findings of this work extend our knowledge of ORF3a, a key accessory protein encoded by SARS-CoV-2, which will probably help to shed light on the pathogenicity of this deadly coronavirus.

## Supplementary information

Supplemental Data

Marked-up version of Maunscript Revised

## References

[1] Qin L, Jeng H, Rakue Y, Mizota T (2005). A deficient public health system as a contributing cause of Severe Acute Respiratory Syndrome (SARS) epidemic in mainland China. Southeast Asian J. Trop. Med. Public Health.

[2] Arabi YM (2015). Severe neurologic syndrome associated with Middle East respiratory syndrome corona virus (MERS-CoV). Infection.

[3] Frieman M, Heise M, Baric R (2008). SARS coronavirus and innate immunity. Virus Res..

[4] Roulston A, Marcellus RC, Branton PE (1999). Viruses and apoptosis. Annu. Rev. Microbiol..

[5] O’Brien V (1998). Viruses and apoptosis. J. Gen. Virol..

[6] Freundt EC (2010). The open reading frame 3a protein of severe acute respiratory syndrome-associated coronavirus promotes membrane rearrangement and cell death. J. Virol..

[7] Law PTW (2005). The 3a protein of severe acute respiratory syndrome-associated coronavirus induces apoptosis in Vero E6 cells. J. Gen. Virol..

[8] Chen M, Wang J (2002). Initiator caspases in apoptosis signaling pathways. Apoptosis.

[9] Minakshi R, Padhan K (2014). The YXXPhi motif within the severe acute respiratory syndrome coronavirus (SARS-CoV) 3a protein is crucial for its intracellular transport. Virol. J..

[10] Chan CM (2009). The ion channel activity of the SARS-coronavirus 3a protein is linked to its pro-apoptotic function. Int. J. Biochem. Cell Biol..

