# Supplementary material for: The ORF3a protein of SARS-CoV-2 induces apoptosis in cells
Source: Cell Mol Immunol. 2020 Jun 18;17(8):881–3. doi: 10.1038/s41423-020-0485-9 (PMC7301057; doi:10.1038/s41423-020-0485-9)
Supplement: Supplementary file 1 — Supplemental Data [file 41423_2020_485_MOESM1_ESM.pdf]

The ORF3a protein of SARS-CoV-2 induces apoptosis in cells

Yujie Ren[1,2,6], Ting Shu[2,3,4], Di Wu[2,3], Jingfang Mu[2,3], Chong Wang[1,2,6], Muhan Huang[2,3], Yang Han[2,3,4], Xue-yi Zhang[2,5], Wei Zhou[1], Yang Qiu[2,3,4,7]*, Xi Zhou[2,3,4,5,6,7]*

Supplementary Data

Supplementary Materials and Methods

Supplementary References

Supplementary Figures 1-2

Materials and Methods

Cell culture and transient transfection

Cell culture and transient transfection were described previously[1]. In brief, Vero E6 cells, HEK393T cells, and HepG2 cells were cultured in DMEM containing 10% FBS (Gibco), 1% streptomycin-penicillin (Gibco) at 37°C in a 5% CO_2 incubator. Cells were seeded onto the dish 24 h prior to transfection, and plasmids were transiently transfected by using TransEasy™ Transfection Reagent according to manufactory's instruction (Forgene).

Plasmid construction

Plasmid construction was described previously[2]. In brief, we got the clones of SARS-CoV and SARS-CoV-2 ORF3a through the combination of whole genes. The clones were digested with restriction enzymes (SalI and NotI, Thermo) and ligated to the PRK-FLAG vector with T4 ligase (Thermo). The wild type PRK-FLAG-ORF3a construct was used as a template to generate the mutant ORF3a constructs. All mutations were confirmed by DNA sequencing.

Cytosol-membrane fractionation assays

Cells seeded on 10 cm dish were transfected with 1 μg plasmid as indicated. 24 h after transfection, cells were washed with PBS and collected by centrifugation at 1500 *g* for 5 min. Then the membrane and plasma proteins were extracted using the "Membrane and Cytosol Protein Extraction Kit (Beyotime)". Briefly, add 1 mL membrane protein extraction reagent A (added with PMSF before use) to cells, gently and fully suspend the cells and place them in an ice bath for 10-15 minutes. Transfer the cell suspension to a homogenization tube and homogenize about 30-50 times. The supernatant was

removed into a new centrifuge tube and then centrifuged to precipitate cell membrane fragments; the supernatant is the plasma protein.

Western blot assays and antibodies

Western blot assays was described previously[3]. In brief, at the indicated times post-transfection, cells were scraped then lysed in cell lysis buffer [20 mM Tris/ HCl (pH 8), 150 mM NaCl, 1% Triton X-100, 1 mM EDTA, 1 mM EGTA, 1 mM NaF, 1 mM sodium orthovanadate and protease inhibitor cocktail (Selleck)] and clarified by centrifugation at 12000 *g* for 15 min at 4°C. The protein concentration of the lysate was incubated with loading buffer for 10 min at 95°C. Then the samples were separated with SDS polyacrylamide gel electrophoresis (SDS-PAGE); Then using the PVDF membrane for membrane transfer. Primary antibodies used as follows: anti-Bid/-tBid (BD Biosciences); anti-cytochrome *c* (Abcam); anti-glutamate dehydrogenase (GDH; CST); HRP-conjugated goat-anti mouse or rabbit IgG (Thermo Scientific); anti-β-Actin (Sigma); anti-caspase-8 (CST)； anti-caspase-9 (CST); anti-Bcl2 (ABclonol Technology); anti-FLAG (Sigma).

Mitochondria fractionation assays

At the indicated times post-transfection, cells were washed with PBS followed by dousing 20 times in 1 mL homogenization buffer (ApplyGen) by 1 mL injector. The homogenate was centrifuged at 500 *g* for 10 min. The supernatant was centrifuged at 5000 *g* for 10 min to precipitate mitochondria.

Immunofluorescence staining assays

Cells seeded on the confocal dish were transfected with 1 μg indicated plasmids. 24 h

after transfection, cells were washed three times with PBS and fixed with 3.7% formaldehyde in PBS for 30 min, then permeabilized by 1% Triton X-100 in PBS for 20 min. After blocking with 5% BSA in PBS for 60 min, cells were incubated with mouse anti-FLAG antibody diluted in 1:100 in 5% BSA in PBS at 4°C overnight. After washing three times with PBS, cells were incubated with Alexa Fluor 488 goat anti-mouse IgG (H+L) (Abcam) diluted in 1:500 in 5% BSA in PBS for 30 min. After washing cells three times with PBS, cell nuclei were stained with DAPI (Invitrogen) diluted in 1:250 in PBS for 5 min. Fluorescent signals were detected by using a Nikon microscope and images were analyzed with the NIS-Elements Viewer 4.50.

Apoptosis detection assays

Flow cytometry assay was used to detect cell apoptosis and analyze the cell cycle[4]. Cells seeded on the dish were transfected with designated plasmids or treated with different caspase inhibitors (Targetmol) or caspase inducers (MCE). After 24h, cells were washed three times with PBS and collected by centrifugation at 1500 *g* for 5 min, following used the "Annexin V-FITC Apoptosis Detection Kit (Beyotime C1062)" to detect cell apoptosis. The operation steps are briefly described as follows: Add 195 μL Annexin V-FITC binding solution to resuspend cells gently; Then add 5 μL Annexin V-FITC and 10 μL propidium iodide staining solution and mix gently; Incubate at room temperature (20-25°C) in the dark for 10-20 minutes, then place in an ice bath; Flow cytometry analysis within one hour.

Caspase-3/7 activity detection assays

Cells seeded on the dish were transfected with designated plasmid. 24 h post-transfection, cells were washed three times with PBS and collected by

centrifugation at 1500 *g* for 5 min. Then use "Caspase-3/7 Green Flow Cytometry (Invitrogen)" to detect cells displayed caspase-3/7 activation. The operation steps are briefly described as follows: Prepare flow cytometry tubes each containing 1 mL of cell suspension; Add 1 μL of CellEvent® Caspase-3/7 Green Detection Reagent (Component A) to samples; incubate for 25 minutes at 37°C; Add 1 μL of the 1 mM SYTOX® AADvanced™ dead cell stain solution to samples; incubate for 5 minutes at 37°C; Analyze the samples using 488-nm excitation and apply for standard fluorescence compensation.

Supplementary References

1 Lu, B. *et al.* Induction of INKIT by Viral Infection Negatively Regulates Antiviral Responses through Inhibiting Phosphorylation of p65 and IRF3. *Cell host & microbe* **22**, 86-98 e84, doi:10.1016/j.chom.2017.06.013 (2017).

2 Ren, Y. *et al.* The Type I Interferon-IRF7 Axis Mediates Transcriptional Expression of Usp25 Gene. *The Journal of biological chemistry* **291**, 13206-13215, doi:10.1074/jbc.M116.718080 (2016).

3 Miao, M. *et al.* Proteomics Profiling of Host Cell Response via Protein Expression and Phosphorylation upon Dengue Virus Infection. *Virologica Sinica* **34**, 549-562, doi:10.1007/s12250-019-00131-2 (2019).

4 Ding, Y. *et al.* Oxymatrine Inhibits Bocavirus MVC Replication, Reduces Viral Gene Expression and Decreases Apoptosis Induced by Viral Infection. *Virologica Sinica* **34**, 78-87, doi:10.1007/s12250-019-00088-2 (2019).

Figure S1

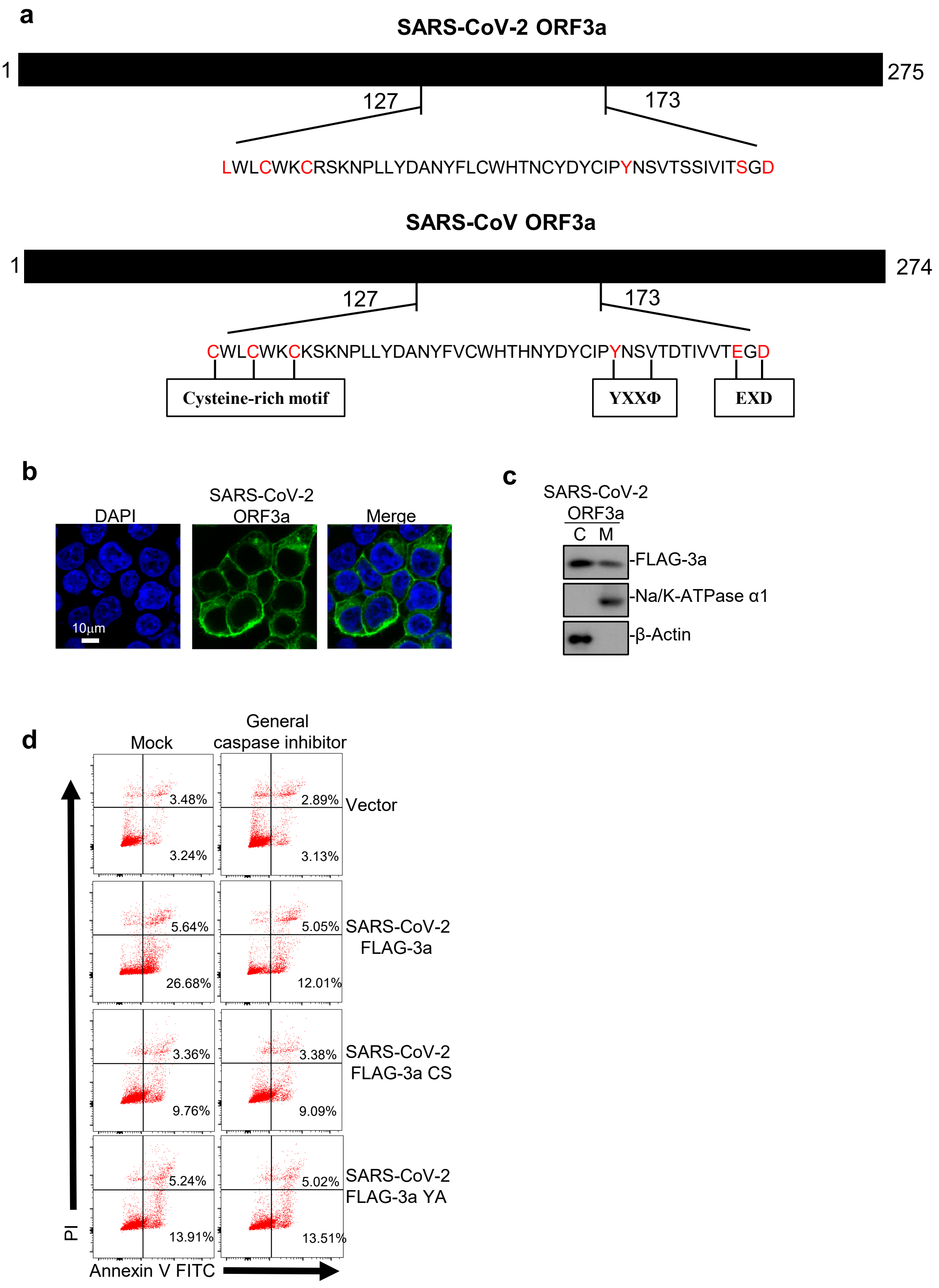

Figure S1. (a) Sequence alignment of SARS-CoV-2 ORF3a and SARS-CoV ORF3a. **(b-c)** HEK293T cells were transfected with FLAG-SARS-CoV-2 ORF3a. After 24h, cells were stained with mouse anti-FLAG antibody and Alexa-488 conjugated anti-mouse IgG for Immunofluorescence (b); To examine the subcellular location of SARS-CoV-2 ORF3a, membrane and plasma proteins were separated, respectively and analyzed via western blotting (c). **(d)** Vero E6 cells were transfected with FLAG-SARS-CoV-2 ORF3a mutants (CS and YA) and treated with DMSO or general caspase inhibitor. After 24h, cells were treated with Annexin V-FITC/ PI for flow cytometry analysis

Figure S2

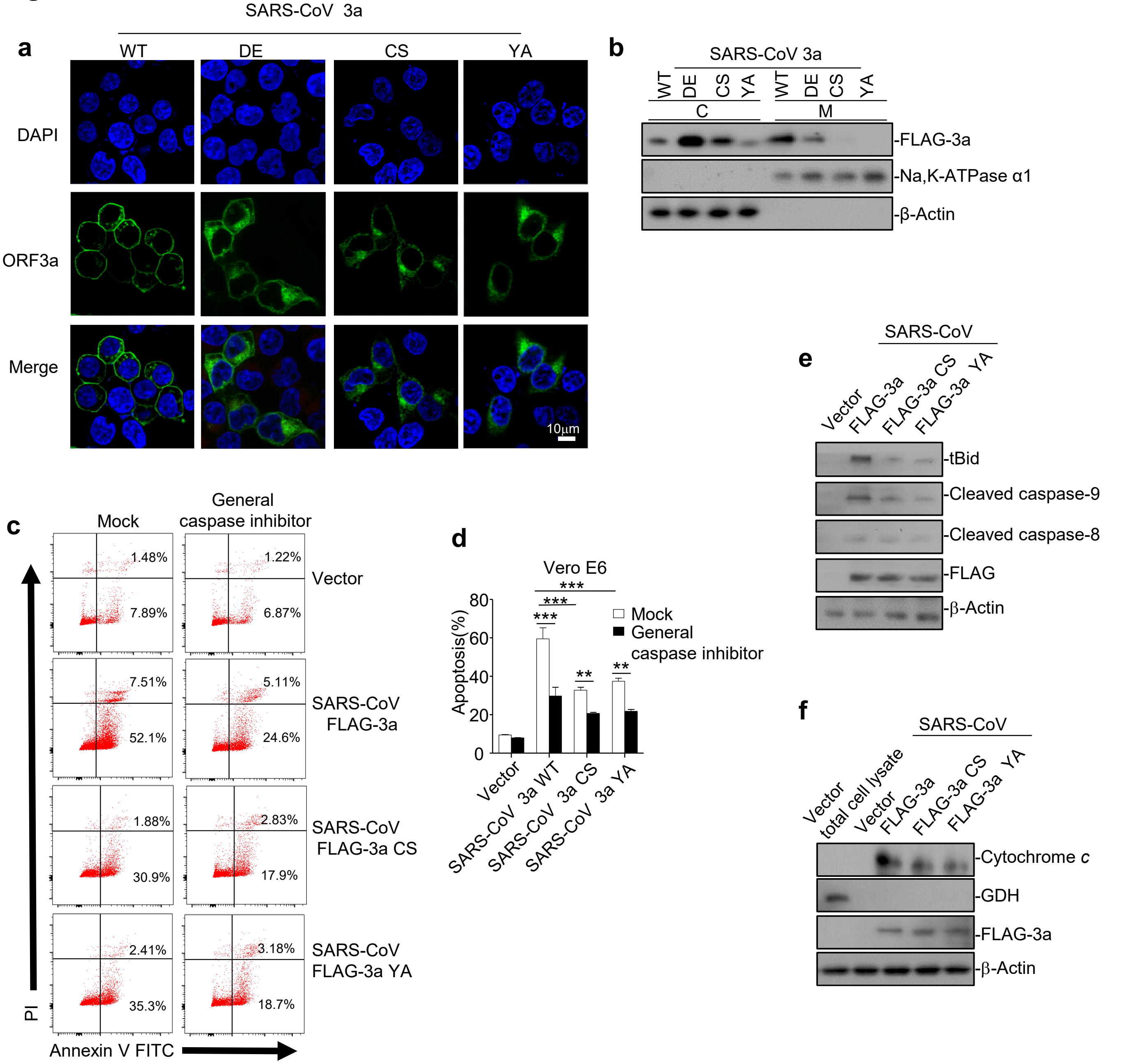

Figure S2. (a) HEK293T cells were transfected with FLAG-SARS-CoV ORF3a and its mutants (DE, CS and YA). After 24h, cells were stained with mouse anti-FLAG antibody and Alexa-488 conjugated anti-mouse IgG for Immunofluorescence. **(b)** HEK293T cells were transfected with FLAG-SARS-CoV ORF3a and its mutants (DE, CS and YA). After 24h, cells were collected and the membrane and plasma proteins were separately extracted for western blotting, respectively. **(c-d)** Vero E6 cells were transfected with FLAG-SARS-CoV ORF3a and its mutants (DE, CS and YA) and treated with DMSO or general caspase inhibitor. After 24h, cells were stained with Annexin V-FITC/ PI for flow cytometry analysis(c), and the percentage of apoptotic cells were measured (d); **(e-f)** HEK293T were transfected with vector or FLAG-SARS-CoV ORF3a and its mutants (DE, CS and YA). After 24h, cells were collected and the proteins were extracted for western blot (e). To examine the levels of cytochrome c in cytosol, mitochondria were separated via gradient centrifugation, and cell lysate without mitochondria were subjected to western blotting (f). **p<0.01, ***p<0.001 by two-tailed Student's t test.
