# Supplementary material for: The ORF3a protein of SARS-CoV-2 induces apoptosis in cells
Source: Cell Mol Immunol. 2020 Jun 18;17(8):881–3. doi: 10.1038/s41423-020-0485-9 (PMC7301057; doi:10.1038/s41423-020-0485-9)
Supplement: Supplementary file 2 — Marked-up version of Maunscript Revised [file 41423_2020_485_MOESM2_ESM.pdf]

The ORF3a protein of SARS-CoV-2 induces apoptosis in cells

Yujie Ren[1,2,6], Ting Shu[2,3,4], Di Wu[2,3], Jingfang Mu[2,3], Chong Wang[1,2,6], Muhan Huang[2,3], Yang Han[2,3,4], Xue-Yi Zhang[2,5], Wei Zhou[1], Yang Qiu[2,3,4,7]*, Xi Zhou[2,3,4,5,6,7]*

删除的内容: Xueyi

[1] Center for Precision Translational Medicine of Wuhan Institute of Virology & Guangzhou Women and Children's Medical Center, Guangzhou Women and Children's Medical Center, Guangzhou, Guangdong, 510120, China

[2] State Key Laboratory of Virology, Wuhan Institute of Virology, Center for Biosafety Mega-Science, Chinese Academy of Sciences (CAS), Wuhan, Hubei, 430071 China

[3] Joint Laboratory of Infectious Diseases and Health, Wuhan Institute of Virology & Wuhan Jinyintan Hospital, CAS, Wuhan, Hubei 430023 China

[4] Center for Translational Medicine, Wuhan Jinyintan Hospital, Wuhan, Hubei 430023 China

[5] State Key Laboratory of Virology, College of Life Sciences, Wuhan University, Wuhan, Hubei 430072 China

[6] Center for Precision Translational Medicine of Wuhan Institute of Virology & Guangzhou Women and Children's Medical Center, Wuhan Institute of Virology, CAS, Wuhan, Hubei 430071 China

[7] University of Chinese Academy of Sciences, Beijing 100049 China

*Correspondence: zhouxi@wh.iov.cn (X.Z.) and yangqiu@wh.iov.cn (Y.Q.)

Severe acute respiratory syndrome coronavirus-2 (SARS-CoV-2) has caused the ongoing pandemic of Coronavirus Disease 2019 (COVID-19). SARS-CoV-2 belongs to the genus *Betacoronavirus* of the *Coronaviridae* family, which includes SARS-CoV and Middle East respiratory syndrome coronavirus (MERS-CoV)[1,2]. Coronavirus-encoded accessory proteins play critical roles in virus-host interactions and the modulation of host immune responses, thereby contributing to coronaviral pathogenicity via different strategies[3]. However, the functions of SARS-CoV-2-encoded accessory proteins are not well understood. Apoptosis is a predominant type of programmed cell death, and has been recognized as an important host antiviral defense mechanism that controls viral infection and regulates the inflammatory response[4,5]. Previous studies have reported that the SARS-CoV-encoded accessory protein ORF3a can induce apoptosis in cells[6,7], leading to the question of whether SARS-CoV-2 ORF3a also has pro-apoptotic activity. Here, we investigated the potential apoptosis-inducing activity of SARS-CoV-2 ORF3a in different cell lines and compared the pro-apoptotic activities of SARS-CoV-2 ORF3a with those of SARS-CoV ORF3a using the same system.

We sought to determine whether SARS-CoV-2 ORF3a can induce apoptosis using annexin V-fluorescein 5-isothiocyanate(FITC)/propidium iodide (PI) double staining in cultured HEK293T, HepG2, and Vero E6 cells. We found that annexin V and PI staining was significantly increased in cells expressing SARS-CoV-2 ORF3a compared to that in control cells (Fig. 1a). Moreover, the quantified data based on measuring the apoptosis rate also confirmed the pro-apoptotic activity of ORF3a in different cell lines (Fig. 1b). Furthermore, we examined activated caspase-3, a marker of caspase-dependent apoptosis, by flow cytometry and found that the percentage of cells with activated caspase-3 was significantly elevated in the presence of ORF3a (Fig. 1c).

删除的内容: The outbreak of severe

删除的内容:),

删除的内容: in

删除的内容: that

删除的内容: modulating

删除的内容: it is barely understood about

删除的内容: .

删除的内容: major

删除的内容: which

删除的内容: any

删除的内容: ,

删除的内容: in

删除的内容: Annexin

删除的内容: Fluorescein

删除的内容:

删除的内容: the

删除的内容: stainings were

删除的内容: those

删除的内容: the

删除的内容: And

删除的内容: for

删除的内容: the activation of

删除的内容: via a

删除的内容: assay by detecting cells with activated caspase-3,

These results show that SARS-CoV-2 ORF3a can efficiently induce apoptosis in cells.

To determine the mechanism through which SARS-CoV-2 ORF3a induces apoptosis, activation of the apoptosis cascade in HEK293T cells expressing ORF3a was examined by western blotting, probing for some apoptosis pathway components at 24 and 48 h post-transfection. Cells treated with staurosporine, an apoptosis inducer, were used as a positive control. SARS-CoV-2 ORF3a induced the cleavage/activation of caspase-8, whereas Bcl-2 expression levels were not affected (Fig. 1d). The cleavage/activation of caspase-8 is recognized as a hallmark of the extrinsic apoptotic pathway, whereas Bcl-2 plays an important role in initiation of the intrinsic pathway[8]. Moreover, we found that the levels of truncated Bid (tBid), cleaved caspase-9, and cytochrome *c* were elevated in the presence of SARS-CoV-2 ORF3a (Fig. 1e), and either a caspase-8 or caspase-9 inhibitor significantly suppressed SARS-CoV-2 ORF3a-induced apoptosis (Fig. 1f-g). Thus, our results imply that SARS-CoV-2 ORF3a can induce apoptosis via the extrinsic pathway, in which activated caspase-8 cleaves Bid to tBid and in turn induces the release of mitochondrial cytochrome *c*, resulting in apoptosome formation and caspase-9 cleavage/activation.

We next sought to examine the relationship between the membrane association and pro-apoptotic activity of SARS-CoV-2 ORF3a. As previously reported, SARS-CoV ORF3a is a transmembrane protein that contains several conserved motifs including a cysteine-rich motif (a.a.127–133), tyrosine-based sorting motif (YXXΦ; a.a.160–163), and diacidic EXD motif (a.a. 171–173), and these domains regulate the subcellular location of SARS-CoV ORF3a and play important roles in SARS-CoV ORF3a infection, inducing apoptosis[9,10]. SARS-CoV-2 ORF3a shares 73% amino acid homology with its counterpart in SARS-CoV, and the cysteine-rich and YXXΦ motifs are conserved but the EXD motif was found to be changed to SGD in SARS-CoV-2

删除的内容: of how
删除的内容: the
删除的内容: via Western
删除的内容: of
删除的内容: h
删除的内容:
删除的内容: (STS),
删除的内容: while
删除的内容: the
带格式的: 字体: 倾斜
删除的内容: inhibitor
带格式的: 字体: 倾斜
删除的内容: the
删除的内容: a
删除的内容:
删除的内容:) (
删除的内容: that
删除的内容: in amino acids
删除的内容: of
删除的内容: is

ORF3a (Fig. S1a). Thus, we constructed two mutant ORF3a proteins by mutating C130/133 of the cysteine-rich motif to S (SARS-CoV-2 ORF3a-CS) or Y160 of the YXXΦ motif to A (SARS-CoV-2 ORF3a-YA). The immunofluorescence assays showed that wild-type ORF3a of SARS-CoV-2 (ORF3a-WT) localized to the plasma membrane with punctate cytoplasmic staining, whereas ORF3a-CS and ORF3a-YA exhibited more cytoplasmic localization (Fig. 1h and S1b). The results of cytosol-membrane fractionation assays showed that whereas ORF3a-WT was present in both cytosol and membrane fractions, either ORF3a-CS or ORF3a-YA was absent in the membrane fraction (Fig. 1i and S1c). Moreover, we found that ORF3a-CS or ORF3a-YA showed minimal apoptosis-inducing and caspase-3-activiting activity in cells in the presence or absence of z-VAD-fmk, a general caspase inhibitor (Fig. 1j and S1d). In addition, ORF3a-CS or ORF3a-YA failed to induce the cleavage of Bid, caspase-8, and caspase-9 or the release of cytochrome *c* (Fig.1k-l). These results indicate that membrane association is required for the pro-apoptotic activity of SARS-CoV-2 ORF3a.

To investigate if there is any difference between the pro-apoptotic activities of ORF3a proteins of SARS-CoV-2 and SARS-CoV, we examined the membrane association and apoptosis-induction ability of SARS-CoV ORF3a. SARS-CoV ORF3a variants were generated by mutating C127/130/133 to S (SARS-CoV ORF3a-CS) in the cysteine-rich motif, Y160 to A (SARS-CoV ORF3a-YA) in the diacidic motif, and 171E/173D to A (SARS-CoV ORF3a-DE) in the EXD motif. We found that SARS-CoV ORF3a-CS and ORF3a-YA mutants were unable to associate with membranes or distribute in membrane fractions, whereas ORF3a-DE still showed membrane association similar to that observed for ORF3a-WT (Fig. S2a-b). Although the ORF3a-CS and ORF3a-YA mutants showed significantly lower apoptosis-inducing and caspase-3-activating capacities than WT ORF3a, they kept some pro-apoptotic

删除的内容: We

删除的内容: a

删除的内容: while

删除的内容: while

删除的内容: showed the presence

删除的内容: activity to induce

删除的内容: activate

删除的内容:

删除的内容: Besides

带格式的: 字体: 倾斜

删除的内容:

删除的内容: mutants

删除的内容: , respectively

删除的内容: while

删除的内容: similarly with

删除的内容: of

删除的内容: mutants

删除的内容: capability to induce

删除的内容: activate

删除的内容: compared with

activities compared to those of the negative control (vector), which could be further inhibited by z-VAD-fmk treatment (Fig. S2c-d). Moreover, the cleavage of Bid, caspase-8, and caspase-9 or the release of cytochrome *c* was apparently reduced but not eliminated in cells expressing SARS-CoV ORF3a-CS or - SARS-CoV ORF3a-YA (Fig. S2e-f). These results indicate that unlike that in SARS-CoV-2 ORF3a, the membrane-association feature is involved in but not essential for the pro-apoptotic activity of SARS-CoV ORF3a (Fig. S3), suggesting that the two ORF3a proteins from different coronaviruses use different strategies to induce apoptosis.

We then sought to compare pro-apoptotic activities between these two coronaviral proteins. Our results showed that compared to those with SARS-CoV-2 ORF3a, SARS-CoV ORF3a expression induced higher levels of apoptosis in Vero E6, HEK293T, and HepG2 cells (Fig. 1m). Consistently, the caspase-3 activation level in SARS-CoV ORF3a-expressing cells was significantly higher than that in cells expressing SARS-CoV-2 ORF3a (Fig. 1n). Therefore, our findings show that SARS-CoV-2 ORF3a has relatively weaker pro-apoptotic activity than SARS-CoV ORF3a. Differences in the pro-apoptotic mechanism and relative strength probably contribute to the differences in pathogenicity between these two coronaviruses (Fig. 1o). Indeed, SARS-CoV-2 has been generally believed to be less virulent than SARS-CoV, and the diminished pro-apoptotic activity of SARS-CoV-2 ORF3a is probably associated with reduced apoptosis-mediated antiviral defence in infected cells. These properties probably confer certain advantages for SARS-CoV-2 in that infection can be relatively mild or even asymptomatic during early stages, thus allowing the virus to spread more widely.

In summary, the findings of this work extend our knowledge of ORF3a, a key accessory protein encoded by SARS-CoV-2, which will probably help to shed light on the pathogenicity of this deadly coronavirus.

删除的内容: with
删除的内容: cleavages
删除的内容: were
带格式的: 字体: 倾斜
删除的内容: feature of
删除的内容:
删除的内容: in apoptotic induction
删除的内容: the
删除的内容: -n
删除的内容: 1o
删除的内容: a
删除的内容: The difference
删除的内容: difference
删除的内容: the
删除的内容: of
删除的内容: S3
删除的内容: less
删除的内容: provide
删除的内容: to
删除的内容: infection and
带格式的: 英语(美国)
删除的内容: Summarily
删除的内容: in
删除的内容: extends
删除的内容: about
删除的内容: ,
删除的内容: sheds

Acknowledgments

This work was supported by the Strategic Priority Research Program of CAS (XDB29010300 to X.Z.), National Science and Technology Major Project (2018ZX10101004 to X.Z.), National Natural Science Foundation of China (81873964 to Y.Q., 31800140 to J.M. and 31670161 to X.Z.), China Postdoctoral Science Foundation (2019M660198 to Y.R.) and Grant from the CAS Youth Innovation Promotion Association (2020332 to Y.Q.).

Author Contributions

Y.R. performed the experiments with the help of T.S., D.W., J.M., W.C., M.H., Y.H. and X.-Y.Z.. W.Z., Y.Q. and X.Z. designed the experiments. Y.R., Y.Q, and X.Z. interpreted the results and wrote the manuscript.

Supplementary Data

Supplementary Materials and Methods

Supplementary References

Supplementary Figures 1-2

删除的内容: figures

删除的内容: 3

REFERENCES

1 Qin, L., Jeng, H., Rakue, Y. & Mizota, T. A deficient public health system as a contributing cause of Severe Acute Respiratory Syndrome (SARS) epidemic in mainland China. *Southeast Asian J Trop Med Public Health* **36**, 213-216 (2005).

2 Arabi, Y. M. *et al.* Severe neurologic syndrome associated with Middle East respiratory syndrome corona virus (MERS-CoV). *Infection* **43**, 495-501, doi:10.1007/s15010-015-0720-y (2015).

3 Frieman, M., Heise, M. & Baric, R. SARS coronavirus and innate immunity. *Virus research* **133**, 101-112, doi:10.1016/j.virusres.2007.03.015 (2008).

4 Roulston, A., Marcellus, R. C. & Branton, P. E. Viruses and apoptosis. *Annu Rev Microbiol* **53**, 577-628, doi:10.1146/annurev.micro.53.1.577 (1999).

5 O'Brien, V. Viruses and apoptosis. *J Gen Virol* **79**, 1833-1845, doi:10.1099/0022-1317-79-8-1833 (1998).

6 Freundt, E. C. *et al.* The open reading frame 3a protein of severe acute respiratory syndrome-associated coronavirus promotes membrane rearrangement and cell death. *J Virol* **84**, 1097-1109, doi:10.1128/JVI.01662-09 (2010).

7 Law, P. T. W. *et al.* The 3a protein of severe acute respiratory syndrome-associated coronavirus induces apoptosis in Vero E6 cells. *J Gen Virol* **86**, 1921-1930, doi:10.1099/vir.0.80813-0 (2005).

8 Chen, M. & Wang, J. Initiator caspases in apoptosis signaling pathways. *Apoptosis* **7**, 313-319, doi:10.1023/a:1016167228059 (2002).

9 Minakshi, R. & Padhan, K. The YXXPhi motif within the severe acute respiratory syndrome coronavirus (SARS-CoV) 3a protein is crucial for its intracellular transport. *Virol J.* **11**, 75, doi:10.1186/1743-422X-11-75 (2014).

10 Chan, C. M. *et al.* The ion channel activity of the SARS-coronavirus 3a protein is linked to its pro-apoptotic function. *Int J Biochem Cell Biol* **41**, 2232-2239, doi:10.1016/j.biocel.2009.04.019 (2009).

Figure legends

Figure 1. (a-b) HEK293T, HepG2, and Vero E6 cells were transfected with FLAG-SARS-CoV-2 ORF3a. After 24 h, cells were stained with annexin V-fluorescein 5-isothiocyanate (FITC)/propidium iodide (PI) for flow cytometric analysis (a), and the percentage of apoptotic cells was measured (b). **(c)** Vero E6 cells were transfected with FLAG-SARS-CoV-2 ORF3a. After 24 h, cells were stained with caspase-3/7 green detection reagent for fluorescence analysis, and the percentage of cells displaying caspase-3 activation was measured. **(d)** HEK293T cells were transfected with empty vector or FLAG-SARS-CoV-2 ORF3a. After 12 and 24 h, cells were subjected to western blotting analysis using the indicated antibodies. Cells treated with STS for 5 h were used as a positive control. STS, staurosporine. **(e)** HEK293T cells transfected with empty vector or FLAG-SARS-CoV-2 ORF3a for 12 and 24 h, or cells treated with STS for 5 h, were collected and the mitochondria were separated via gradient centrifugation. Cell lysates without mitochondria were subjected to western blotting using the indicated antibodies. The total cell lysates within intact mitochondria were used as positive the control. GDH, glutamate dehydrogenase. **(f-g)** Vero E6 cells were transfected with FLAG-SARS-CoV-2 ORF3a in the presence of DMSO, caspase-8 inhibitor, or caspase-9 inhibitor. After 24 h, cells were stained with annexin V-FITC/PI for flow cytometric analysis (f), and the percentage of apoptotic cells was measured (g). **(h)** HEK293T cells were transfected with FLAG-SARS-CoV-2 ORF3a and its mutants (CS and YA). After 24 h, cells were stained with a mouse anti-FLAG antibody and Alexa-488 conjugated anti-mouse IgG for immunofluorescence. Scale bar, 10 μM. **(i)** HEK293T cells were transfected with FLAG-SARS-CoV-2 ORF3a and its mutants (CS and YA). After 24 h, cells were collected and the membrane and plasma proteins were separately extracted for western blotting. **(j)** Vero E6 cells were transfected with FLAG-SARS-CoV-2

删除的内容: 24h
删除的内容: Annexin
删除的内容: /
删除的内容: cytometry
删除的内容: were
删除的内容: 24h
删除的内容: Caspase
删除的内容: displayed
删除的内容: were
删除的内容: .(
删除的内容: 12h
删除的内容: 24h
删除的内容: into
删除的内容: used
删除的内容: 5h
删除的内容: 12h
删除的内容: 24h
删除的内容: , respectively.
删除的内容: lysate
删除的内容: used
删除的内容: lysate
删除的内容: 24h
删除的内容: Annexin
删除的内容:
删除的内容: cytometry
删除的内容: were
删除的内容: 24h
删除的内容: Immunofluorescence
删除的内容: 24h
删除的内容: , respectively

ORF3a mutants (CS and YA) and treated with DMSO or a general caspase inhibitor. After 24 h, cells were treated with annexin V-FITC/PI for flow cytometric analysis, and the percentage of apoptotic cells was measured. **(k-l)** HEK293T cells were transfected with vector or FLAG-SARS-CoV-2 ORF3a and its mutants (CS and YA). After 24 h, cells were collected and the membrane and plasma proteins were separately extracted for western blotting (k). To examine levels of cytochrome *c* in the cytosol, mitochondria were separated via gradient centrifugation, and cell lysates without mitochondria were subjected to western blotting (l). **(m)** HEK293T, HepG2, and Vero E6 cells were transfected with vector, FLAG-SARS-CoV ORF3a, or FLAG-SARS-CoV-2 ORF3a. After 24 h, cells were stained with annexin V-FITC/PI for flow cytometric analysis, and the percentage of apoptotic cells was measured. **(n)** Vero E6 cells were transfected with vector, FLAG-SARS-CoV ORF3a, or FLAG-SARS-CoV-2 ORF3a. After 24 h, caspase-3 activation was detected by using caspase-3/7 green detection reagent, and the percentage of cells displaying caspase-3 activation was measured. **(o)** Left, the pro-apoptotic activity of SARS-CoV-2 ORF3a requires the membrane association of ORF3a. Right, membrane association is involved but not essential for the pro-apoptotic activity of SARS-CoV ORF3a, and SARS-CoV ORF3a can induce apoptosis in a membrane-independent manner. SARS-CoV-2 ORF induces apoptosis in a lesser extent than that of SARS-CoV ORF3a. $^{**}p < 0.01$, $^{***}p < 0.001$ by two-tailed Student's *t* test.

删除的内容: 24h
删除的内容: Annexin
删除的内容:
删除的内容: cytometry
删除的内容: were
删除的内容: 24h
删除的内容: blot, respectively (
删除的内容: the
带格式的: 字体: 倾斜
删除的内容: lysate
删除的内容: -n
删除的内容: , respectively.
删除的内容: 24h
删除的内容: Annexin
删除的内容:
删除的内容: cytometry
删除的内容: (m),
删除的内容: were
带格式的: 字体: 加粗, 图案: 清除
删除的内容:). (o
删除的内容: 24h
删除的内容: were
删除的内容: Caspase
删除的内容: displayed
删除的内容: were
带格式的: 字体: 加粗
删除的内容: <
删除的内容: <
